# Screening for Neurocognitive Impairment in HIV-Infected Individuals at First Contact after HIV Diagnosis: The Experience of a Large Clinical Center in Northern Italy

**DOI:** 10.3390/ijms17040434

**Published:** 2016-03-24

**Authors:** Emanuele Focà, Paola Magro, Davide Motta, Silvia Compostella, Salvatore Casari, Andrea Bonito, Nigritella Brianese, Alice Ferraresi, Paola Rodari, Maria Chiara Pezzoli, Eugenia Quiros-Roldan, Francesco Castelli

**Affiliations:** Department of Infectious and Tropical Diseases, University of Brescia and Brescia Spedali Civili General Hospital, Piazzale Spedali Civili 1, 25123 Brescia, Italy; p.magro@unibs.it (P.M.); davide.motta84@gmail.com (D.M.); compostar@hotmail.com (Si.C.); s.casari@infettivibrescia.it (S.C.); andreabonito@hotmail.com (A.B.); nigri_bri@libero.it (N.B.); alice-ferraresi@hotmail.it (A.F.); paolarodari@icluod.com (P.R.); chiarapezzoli@yahoo.com (M.C.P.); eugeniaquiros@yahoo.it (E.Q.-R.); francesco.castelli@unibs.it (F.C.)

**Keywords:** HIV, neurocognitive disorders, HIV-associated dementia

## Abstract

Neurocognitive disorders are emerging, probably underestimated, complications in HIV-infected people. The aim of the study was to assess neurocognitive profiles of newly detected HIV-infected patients. We performed an observational retrospective single-cohort study. Illiterates and patients with neurologic symptoms or previous psychiatric diagnosis were excluded. Neuropsychological profiles were assessed using a validated battery of neuropsychological tests. We included 206 patients; with males representing the majority of them (85%). Risk factors for HIV acquisition were unprotected sexual intercourse (homo/bisexual in 39.8% and heterosexual in 60.2%). Thirty-nine patients (18.9%) were previous injection drug users, while 41 (19.9%) were alcohol abusers. Mean education was 11.1 years (SD—standard deviation—3.7). A high prevalence of HIV-associated neurocognitive disorders (HAND, 47.1%) was present in HIV-infected patients: particularly, asymptomatic neurocognitive impairment (ANI) was found in 30.6%, mild neurocognitive disorder (MND) in 15% and HIV-associated dementia (HAD) in 1.5%. Male gender, low degree of education, AIDS diagnosis and gepatitis B virus (HBV) co-infection were factors independently associated with HAND in a multivariable logistic regression model. Our data suggest that patient-specific factors and AIDS diagnosis have a certain kind of impact in HAND occurrence. A complete neuropsychological screening must be recommended in all patients at HIV-infection diagnosis.

## 1. Introduction

Since the beginning of the HIV/AIDS epidemic, neurologic manifestations associated with HIV-infection have been an important cause of morbidity in HIV-infected individuals [1].

In fact, the incidence of HIV-associated Dementia (HAD) within two years from the diagnosis of HIV-infection used to be of 7% in the early 1990s [2]. More recently, thanks to the introduction of highly active antiretroviral therapy (HAART), the incidence of HAD has drastically diminished [3]. Despite this important result, the spectrum of less severe forms of HIV-associated neurocognitive disorders (HAND), including asymptomatic neurocognitive impairment (ANI) and mild neurocognitive disorders (MND), seems to be increasing [4]. The causes of this phenomenon can be various, and are still not entirely understood [5].

Various risk factors have been associated with a higher prevalence of HAND, including host factors (genetic predisposition, cardiovascular risk factor, drug addiction or aging), HIV-related factors (AIDS, drug resistance or neurotoxicity, neuroinflammation) and co-morbidities hepatitis C virus (HCV, depression, obesity, diabetes) [6].

In several studies [7,8,9], lower nadir CD4^+^ T-cell counts were significantly associated with the onset of neurocognitive impairment in HIV-infected patients. A possible pathogenetic mechanism suggested could be that a lower nadir of CD4^+^ T-cells may allow a greater entrance of viruses inside the Central Nervous System (CNS) [10].

However, other studies did not find this association. A correlation between the presence of HAND and high viral plasmatic levels is even less clear in the HAART era [11,12].

Despite its unquestionable beneficial effects in the struggle against HIV-infection, HAART itself has been ascribed as a possible factor of neuro-damage [13,14]. Neurotoxicity of HAART could be due to both direct (mithocondrial toxicity [15], proteosomical dysfunctions [16]) and indirect mechanisms (lypodistrophy and resulting increasing risk of cerebrovascular disease) or to host genetic factors (decreased metabolism of efavirenz due to genetic variants [15]). However, different studies do not support this hypothesis. In these studies, patients with a longer duration of HAART tended to have better cognitive achievements and a lower incidence of HAND [11,17].

A question that still goes unanswered is whether a HAART regimen with a higher penetration rate into the CNS can lead to better neurocognitive performances in HIV infected patients [18]. In the revised 2010 version of the CNS Penetration-Effectiveness (CPE) score proposed by the CHARTER group in the USA [19], a score from one to four (where four is the most neuroeffective drug) has been attributed to antiretroviral drugs depending on their pharmacokinetic and pharmacodynamic characteristics. The use of HAART regimens with a higher CPE score has been associated with lower viral load in the cerebrospinal fluid (CSF), although not always linked with better neurocognitive performances [20].

Currently, prevalence rates of HAND, usually evaluated in HAART experienced HIV-positive populations were recently reported to be higher [11] than previously thought [17,21,22].

A recent European multicenter study from Winston *et al.* [23] explored factors associated with cognitive function in HAART-naïve HIV-infected subjects with relatively well-preserved immunity and early HIV infection at the time of enrollment in the NEAT 001/Agence Nationale de Recherche sur le SIDA (ANRS) 143 study. Factors significantly and independently associated with poorer overall cognitive performance were older age, shorter duration of education, black ethnicity, lower height, and, unexpectedly, lower plasma HIV RNA.

In order to confirm these findings, particularly among those individuals who had received their first HIV-infection/AIDS diagnosis, we assessed neurocognitive profiles in these kinds of patients as routine clinical practice.

The aim of our study was to evaluate clinical and socio-demographical factors possibly associated with neuropsychological performances in a “real life” setting, irrespective of the viro-immunological state of the patient at the moment of the enrollment in our study.

## 2. Results

### 2.1. Patients’ Characteristics

As many as 206 patients were included in the study (Table 1): 175 (85%) were males and mean age was 40.2 (SD—standard deviation—10.4) years. As risk factors for HIV acquisition, respectively 39.8% and 60.2% of patients declared homo/bisexual and heterosexual intercourse, while 18.9% were injection drug users. Only 17 patients (8.3%) were co-infected with HCV; hepatitis B virus (HBV) infection was present in 29 cases (14.1%). Twenty-one patients (10.2%) were taking psychiatric drugs, mostly mild anxiolytic or antidepressant medications; 41 patients (19.9%) were alcohol abusers. Overall, patients received a mean of 11.1 years (SD 3.7) of education.

### 2.2. Neurocognitive Disorders

Globally, HAND were present in 97 (47.1%) patients; in particular, asymptomatic neurocognitive impairment (ANI) was found in 63 (30.6%) patients, whereas 31 (15%) and three (1.5%) patients were diagnosed with mild neurocognitive disorder (MND) and HIV-associated dementia (HAD), respectively.

Furthermore, 18 patients (8.7%) resulted in having a low score only on the mini mental state examination (MMSE) test, not falling within the definition of any of the HAND categories, while 73 patients (36.9%) were found to be depressed, according to their scores in the Zung Self-Rating Depression Scale. We performed an adjunctive analysis in order to explore the correlation between low score at the Zung’s test and assumption of antidepressant and we found a correlation at the Pearson test (*p* = 0.001).

### 2.3. Factors Associated with HIV-Associated Neurocognitive Disorders (HAND)

Logistic regression analysis to assess baseline factors possibly associated with HAND (Table 2) showed, in the stepwise backward approach, that male gender (HR 0.38: 95% CI 0.15–0.95, *p* = 0.04), lower degree of education (HR 0.85; 95% CI 0.78–0.93; *p* = 0.001), presence of HBV co-infection (HR 2.94; 95% CI 1.13–7.63; *p* = 0.026) and a history of an AIDS-defining disease (HR 3.36; 95% CI 1.35–8.35; *p* = 0.009) were independently associated with HAND presentation.

## 3. Discussion

In this cohort of HIV-infected patients, male gender, low degree of education, a history of an AIDS-defining disease and HBV co-infection were factors independently associated with the presence of HAND.

### 3.1. HAND Prevalence and Classification

We found an overall prevalence of HAND of 47.1% in our population. Asymptomatic neurocognitive impairment (ANI) was diagnosed in 63 (30.6%) patients, whereas 31 (15%) had mild neurocognitive disorder (MND) and three (1.5%) met criteria to be diagnosed as having HIV-associated dementia (HAD). These findings are somehow homogeneous with other studies [6,23].

In the cohort examined by Simioni *et al.* [11], a higher rate of HAND was found (69%). Anyway, in the aforementioned study, 50% of the patients enrolled were already complaining a certain degree of cognitive impairment, thus probably presenting an already more advanced stage of neurologic decline.

Nowadays, ANI and MND are more common than HAD, both in treatment-naïve and in experienced patients. Some studies presented a higher prevalence of ANI than MND, while others report the opposite trend [6]. In the current study, the most represented category of HAND was ANI. Although the role of this diagnostic category has been questioned [24], it is true that a certain degree of neurocognitive deterioration can go unobserved for a long period, and so not brought to the attention of the physician by the patient. Since neurocognitive impairment can remarkably undermine the quality of life of these patients, screening for neurocognitive performance of HIV-infected patients at the moment of the first diagnosis of HIV-infection could be a precious tool for an early diagnosis and a prompt recognition of the presence of HAND. Regrettably, the administration of a complete battery of neuropsychological tests requires spending a significant amount of time, and the presence of qualified personnel, making a complete evaluation of the neurocognitive profile not always possible in a clinical setting.

### 3.2. Cortical Profile and Depression Grade

We observed that 18 patients were found to have a low score only in the MMSE, although not falling within the criteria to be diagnosed for HAND. Although MMSE has been found to be a weak tool for diagnosing HAND [25], it has been used in our study as a screening instrument for a more complete neurocognitive assessment of the patients at baseline.

A low score on this test could be interpreted as a warning signal of a different kind of involvement, typical of the cortical impairment observed, for instance, among patients affected by Alzheimer-like diseases. On the other hand, a less severe degree of impairment of the CNS may be present in a subset of individuals. Therefore, a re-evaluation of neurocognitive profile should be recommended in these patients.

In addition, 73 patients (36.9%) were found to suffer from depression after being evaluated with the Zung Self-Rating Depression Scale. None of our patients, at the moment of enrollment, had a previous formal psychiatric diagnosis, although 10.2% of them were treated with mild antidepressants or an anxiolytic, and we found a correlation between these drugs and a low score on Zung’s self-reported test. This result may reflect, in our patients, the presence of an understandable degree of concern and anxiety due to the recent diagnosis of HIV-infection. As revealed in another study [26], depression may be underdiagnosed in the HIV-infected population. Whether depression is associated or not with a worse neurocognitive outcome in these kind of patients is yet to be found. No association was found in ours nor in the abovementioned study. Nevertheless, prevalence of suicide has been observed to be higher in HIV-infected patients compared to the general population [27], thus an early detection of depression may possibly prevent a fatal outcome, and a specific treatment may be provided.

### 3.3. Risk Factors Associated with HAND

As with other clinical trials [23,26], traditional demographic risk factors, such as older age and lower degree of education, were found to be associated with a worse neurocognitive performance. As in the study from Winston *et al.* [23], no association was found between the viro-immunological status and the presence of HAND. Anyway, while in the main NEAT-001 trial a <500 cell/ µL cut-off of the CD4^+^ T-cells had been settled, in our study the value of CD4^+^ T-cell count didn’t represent an exclusion /inclusion criteria, therefore allowing us to evaluate a more heterogeneous population.

Co-infection with HBV and HCV viruses is a frequent condition in the HIV population. All of these viruses can invade the CNS, and co-infected patients are more likely to develop a certain degree of neurocognitive impairment compared to mono-infected patients [28]. In fact, we found an independent correlation between presence of HBV co-infection and worse performances on neurocognitive tests.

Male gender was also found to be a risk factor for the development of HAND, perhaps reflecting a greater resilience or the existence of protective factors in women. It is also true that patients included in our study were largely males, and, therefore, this could affect the significance of this result.

In the current study, as in other research [26], we also found that presence of HAND was more frequent in elderly patients, even if this factor was not associated with HAND in logistic regression analysis. Both immunosenescence and a greater duration of the disease are important factors in HAND presentation. As long as HAART is remarkably increasing life expectancy of HIV-infected individuals, a growing concern arises about the possibility of a greater incidence of neurocognitive impairment in elderly HIV-infected patients when compared to the age-matched general population [29]. In a previous study realized in our Center [30], we observed a worse cognitive performance in elderly HIV-infected women when compared to age-matched HIV-negative women. These patients presented a subcortical cognitive profile of executive dysfunction, slowing speed of processing, and deficient verbal learning, even if virologically suppressed. Therefore, aging patients should undergo periodic re-evaluations in order to identify an early deterioration of cognitive functions.

A history of diagnosis of an AIDS-defining pathology independently correlates with the occurrence of neurocognitive deterioration, thus perhaps reflecting a more compromised general status. These findings used to be more frequent, particularly in the pre-HAART era, being more rarely observed nowadays [2,3].

The present study suffers from several limitations that have to be discussed. First, the lack of follow-up data did not allow us to perform a longitudinal evaluation of the evolution of neurocognitive disorders and the effect that antiretroviral therapy has on them. Second, due to the observational nature of this study we had a very heterogeneous population. This could be, however, a trustworthy image of a population possibly met in a “real life” clinical setting. Lastly, it was not possible for us to validate our neuropsychological data through comparing them by neuroimaging or lumbar puncture results.

## 4. Experimental Section

### 4.1. Materials and Methods

#### Subjects

We performed an observational, retrospective, single-cohort study. We enrolled patients with documented HIV-infection at the time of their first evaluation at the University Department of Infectious and Tropical Diseases of University of Brescia and Spedali Civili General Hospital (Brescia, Italy). Patients were enrolled between January 2009 and May 2013.

All patients were >18 years old and with a confirmed HIV infection; moreover, to be included in the study, patients had to provide a valid informed consent and HAART could not have started more than 40 days before. Exclusion criteria were the presence of previous CNS opportunistic infections, neurologic or psychiatric diseases, as well as current assumption of psychiatric drugs that could interfere with cognitive performances (mild anxiolytic or antidepressant drugs were permitted) and cultural or linguistic inability to undergo planned investigations.

### 4.2. Standard Protocol Approvals, Registration and Patient Consents

These procedures were approved by the Ethical Committee of Brescia Spedali Civili General Hospital on 03 September 2013. Written informed consents were obtained from all the patients involved in the study.

### 4.3. Patient Recruitment

#### Procedures

A medical, behavioral and neuropsychological evaluation has been performed for every patient at the first contact after HIV-infection diagnosis.

### 4.4. Neuropsychological Examination

All the patients involved in the study underwent the following array of neuropsychological tests. Several cognitive functions, including verbal and visuo-spatial memory, attention, frontal functions, capability of abstracted reasoning and language, have been evaluated:

- Visuo-spatial ability and memory: Rey–Osterrieth Complex Figure Test (ROCF), Copy and Delayed Recall;
- Verbal memory: Short Story, Digit Span Forward;
- Verbal fluency: Fluency for Letter, Fluency for Category;
- Executive functions and attention: Trail Making Test A e B.

Two more broad-spectrum questionnaires have been added to this array of essentially neuropsychological tests:

- The MMSE has been used as a screening test, to evaluate possible disorders of intellectual efficiency and the presence of intellectual deterioration. This test examines eight different cognitive areas: orientation to time, orientation to space, registration, attention and calculation, recall, language, repetition, complex commands.
- Zung Self-Rating Depression Scale is a brief self-administered questionnaire that has been used to probe the presence of depression in the enrolled patients.

### 4.5. HAND Nosology

According to the updated research nosology for HIV-associated neurocognitive disorders [31], patients have been diagnosed as having ANI when the score obtained on at least two neuropsychological tests exploring two different cognitive areas resulted in at least 1 Standard Deviation (SD) below the mean, without any interferences in the activities of daily living (ADLs). A diagnosis of Mild Neurocognitive Disorder has been established for those presenting a score at least 1 SD below the mean on at least two neuropsychological tests exploring two different cognitive areas, which had mild interference on the ADLs. Finally, those that had performances of at least 2 SD below the mean on at least two neuropsychological tests exploring two different cognitive areas,have been diagnosed as having HIV-Associated Dementia (HAD).

All neuropsychological tests were scored according to reference tables that correct for age, sex and education on the basis of normative data available for Italian population.

### 4.6. Statistical Analysis

Continuous variables were expressed as mean and standard deviation and compared with the Student’s *t*-test. Categorical variables were expressed as numbers and percentages and compared with the Pearson’s χ-squared test or Fisher exact test as appropriated. Ordinal variables were compared with the Mann–Whitney *U* test. Multivariable logistic regression analyses including all the statistically significant results (gender, age, history of AIDS, HBV and/or HCV co-infection and degree of instruction) and variables of clinical interest (CD4^+^ T-cell count, HIV RNA, history of IVDU) were performed in order to determine possible predictors and factors associated with HAND; the semi-automatic stepwise backward approach, with progressive elimination of non-significant variables, was applied to find the model that best fitted with the data. The statistic software SPSS (IBM Corp. Released 2010. IBM SPSS Statistics for Windows, Version 19.0. Armonk, NY: IBM Corp, Armonk, NY, USA) was used, and the *p* value was two-tailed and considered significant when <0.05.

## 5. Conclusions

In conclusion, our study provided new data from routine clinical practice in the current effort of assessing HIV-associated neuropsychological disturbances. Our data confirm that a complete and validated battery of neuropsychological tests must be warranted as a screening tool on the first evaluation after diagnosis of HIV-infection in all HIV-infected patients. Patients with male gender and a low grade of instruction may be most at risk for neurocognitive disorders. Moreover, a diagnosis of an AIDS-defining disease and HBV/HCV co-infection must be considered as additional independent risk factors for worse neurocognitive performances. Further studies are required in order to find more accessible tools for the screening of neurocognitive disorders and to compare the results of neuropsychological tests with other techniques.

## Figures and Tables

**Table 1:** Demographic, clinical and neuropsychological characteristics of patients at the baseline.

Variable	Total (*n* = 206)	Patients with HAND (*n* = 97)	Patients without HAND (*n* = 109)	*p* Value
Male (%)	175 (85)	88 (90.7)	87 (79.8)	0.03
Age. years. Mean (±SD)	40.2 (10.4)	42.4 (10.2)	38.2 (10.2)	0.03
Median (IQR)	39 (33–48)	39 (33–47.5)	39 (33–47.2)	
Risk factors:				n.s.
heterosexual intercourse (%)	124 (60.2)	64 (66.0)	60 (55.0)	n.s.
homo/bisexual intercourse (%)	82 (39.8)	33 (34.0)	49 (45.0)	n.s.
injection drug users (%)	39 (18.9)	21 (21.6)	18 (16.5)	n.s.
Co-infection				
HBV (%)	29 (14.1)	21 (21.6)	8 (7.3)	0.03
HCV (%)	17 (8.3)	12 (12.4)	5 (4.6)	0.04
Psychiatric medications (%)	21 (10.2)	15 (15.5)	6 (5.5)	0.02
Alcoholic abuse (%)	41 (19.9)	23 (23.7)	18 (16.5)	n.s.
Education years Mean (±SD)	11.1 (3.7)	10 (3.2)	12 (3.9)	0.001
Median (IQR)	11 (8–13)	11 (8–13)	11 (8–13)	
CD4^+^ T-cell/µL Mean (±SD)	370 (276)	331 (274)	405 (275)	0.054
Median (IQR)	321 (163–545)	304 (151–537)	301 (148–535)	
HIVRNA copies/mL Mean (±SD)	112,566 (289,971)	130,212 (358,380)	96,861 (211,916)	n.s.
Median (IQR)	15,095 (1377–90,668)	12,255 (1044–82,485)	11,080 (1042–78,135)	
CDC class C (%)	29 (14.1)	21 (21.6)	8 (7.3)	0.003
MMSE low score (%)	18 (8.7)	14 (14.4)	4 (3.7)	0.006

HAND: HIV-associated neurocognitive disorders; HBV: hepatitis B virus; HCV; hepatitis C virus; IQR: Interquartile range; CDC: Center for Diseases Control and Prevention; MMSE: mini mental state examination; n.s.: no significative.

**Table 2:** Multivariable logistic regression of baseline risk factors for HIV-Associated Neurocognitive Disorders (HAND) presentation (stepwise backward approach).

Variable	HR	95% CI	*p* Value
Gender: female *vs.* male	0.38	0.15–0.95	0.04
Education years (×1 year increase)	0.85	0.78–0.93	0.001
HBV Co-infection (yes *vs.* no)	2.94	1.13–7.63	0.026
HCV co-infection (yes *vs.* no)	2.80	0.84–9.36	0.093
CDC class 3 (yes *vs.* no)	3.36	1.35–8.35	0.009

HR: hazard ratio; 95% CI: 95% confidence interval; HBV: hepatitis B virus; HCV; hepatitis C virus; IQR: Interquartile range; CDC: Center for Diseases Control and Prevention.
